# NEWS

**DOI:** 10.7189/jogh.03.010201

**Published:** 2013-06

## AFRICA

• Recent statistics on economic growth in Africa have prompted headlines that “Africa is set to become the next big global economic power”. However, analysing Africa’s development prospects based solely on indicators such as GDP growth rates, per capita incomes, or increase in mobile phone use is misleading. Africa is still deficient in terms of manufacturing. Agriculture and extraction of natural resources are still at the core of most African economies, in contrast to the industrialization that is characteristic of wealthier global economies, such as the USA and China. Indeed, a recent UN report paints a more realistic portrayal of Africa’s development prospects, finding that despite some improvements, some African countries are at a standstill in terms of industrialization, with some even moving backwards. Service industries alone cannot, in the long–term, create enough jobs for the millions of African youth; instead, trade arrangements and bilateral agreements should be revised such that Africa is able to adopt sustainable, long–term industrialization policies. (*The Foreign Policy*, 4 Jan 2013)

• According to the World Health Organization, the Ministry of Health of Chad has launched an emergency mass–vaccination campaign against yellow fever, following laboratory confirmation of two cases in the country in December 2012. The campaign is supported by the Chad’s Ministry of Health, the International Coordinating Group on Yellow Fever Vaccine Provision (YF–ICG11), and GAVI Alliance. (*PANA*, 14 Feb 2013)

• Médecins Sans Frontières/Doctors Without Borders (MSF) teams have completed a cholera vaccination campaign in and around the refugee camps in Maban County in South Sudan – a total of 132 500 persons. This action should limit the spread of an outbreak of cholera, but efforts are still required to improve water and sanitation in the camps. With the cooperation of the South Sudan Ministry of Health, MSF launched the vaccination campaign as part of a cholera preparedness and prevention plan. (*MSF*, 24 Feb 2013)

• The report by FEWSNET – a famine early warning system – estimated that the famine in Somalia in 2011 may have killed up to 260 000 people, half of them pre–school children. The aid community believes that tens of thousands of people died needlessly because the international community was too slow in their response to hunger in East Africa in late 2010 and early 2011. Previous estimates said that between 50 000 and 100 000 people died in the famine crisis. (*Associated Press*, 29 Apr 2013)

• The Democratic Republic of the Congo is the most dangerous place in the world to give birth, with women having one in 30 chance of dying as a result. Finland is the safest, with the risk of one in about 12 000 births. For the past 14 years the “Save the Children” mothers’ index records both the risk of maternal mortality and the difficulties women face when they do become mothers. The bottom countries are predominantly in sub–Saharan Africa and they also include Nigeria, Gambia and Somalia, where one in seven children still die before reaching their fifth birthday, compared to one in about 350 in Finland. This year’s report presents a separate index on newborn deaths for the first time. It has been noted that the mortality of the children under five years has been steadily decreasing globally in recent years, but there has been little progress on newborns in comparison. (*The Guardian*, 7 May 2013)

## ASIA

• The polio vaccination campaign in Pakistan faced fresh setbacks when at least 8 health workers were killed in December 2012. Multiple attacks across different locations saw the campaign temporarily suspended. In the past the campaign has faced considerable resistance from the Taliban, who have denounced the vaccination campaign as a cover for espionage. Polio, a highly infectious disease capable of causing permanent paralysis within hours, remains endemic only in Afghanistan, Nigeria and Pakistan. Officials have highlighted the solid gains made against the disease and the potential for polio to be eradicated in Pakistan. The attacks have been condemned by UNICEF, the World Health Organisation, GAVI Alliance and the government of Pakistan. (*GAVI Alliance*, 19 Dec 2012)

• World Health Organization warned that the shortage of medicines is becoming critical in Syria, where the conflicts between rebels and government forces persist. Hospitals are in severe need of anaesthesia, antibiotics, serums and other essential medicines, with local pharmacies increasingly unable to provide basic medicines. WHO added that the ongoing conflict continues to impact waste management and the availability of safe water (*UN News*, 1 Feb 2013)

• The GAVI Alliance and the Islamic Development Bank (IDB) entered into a partnership with the goal to vaccinate at least 400 million children by 2020 in the bank’s member countries. IDB will help GAVI to secure funds for immunization and an estimated US$ 7 billion would be needed to reach its target under the partnership. IDB Chairman Ahmad Mohamed Ali, who signed the memorandum of understanding with GAVI CEO Seth Berkley, said the bank would help select member countries to implement GAVI’s vaccination program. (*Devex*, 12 Mar 2013)

• A new virus called novel coronavirus (NCoV), which emerged in the Middle East last year killing five people, is adapted to infecting humans but seems treatable with drugs that boost the immune system. The virus is from the same family as the common cold and Severe Acute Respiratory Syndrome (SARS). Up to mid–February there have been 12 confirmed cases worldwide – including in Saudi Arabia, Jordan and Britain. Health officials in the UK found evidence that a new viral illness can spread from person to person. Cases of the infection can mostly be linked to contact with animals, but one person in the UK is thought to have caught the infection from a relative. The new virus is not the same as SARS, but similar to it and also to other coronaviruses found in bats. (*Reuters*, 2 May 2013)

• According to Red Cross, health care workers and health institutions came under violent attack in 22 countries last year, in nearly 1000 violent incidents that included 150 killings. Such violence deprived millions of people in need of care. Apart from killings, the incidents include threats and kidnappings. Mr Pierre Gentile, who heads ICRC’s “Health Care in Danger” project, said that those figures were just “the tip of the iceberg”, because most incidents go unrecorded. (*AFP*, 16 May 2013)

## AUSTRALIA AND WESTERN PACIFIC

• Researchers in Australia have provided a final clue required for developing a new anti–malarial drug, killing the parasite with a salt overdose. The drug could become the first discovery in the fight against malaria in two decades. The malaria parasite survives in red blood cells, which are full of salt, and “...the parasite is quite leaky, but it’s got a very effective molecular salt pump, that keeps pushing the salt out again,” said Professor Kiaran Kirk, director at the Research School of Biology at Australia National University (ANU). Research teams in the United States and Singapore had developed a drug that attacked the protein that makes up the salt pump, but it wasn’t until the ANU researchers tested it that they confirmed it worked effectively. The drug attacks the salt pump and disables it, causing the parasite to fill up with salt and die. Targeting such a basic function is crucial because malaria tends to evolve quickly, developing resistance and rendering other drugs ineffective. (*Reuters*, 19 Feb 2013)

• A report released by the Australian National Health Performance Authority (NHPA) showed that the percentage of children fully immunized has dropped to a disturbing level. The data from the Childhood Immunization Register showed that about 8% of Australian children are not fully immunized, and the Australian Medical Association (AMA) warned that the vaccination rate below 93% is unsafe for protecting the children and the community. AMA’s President, Dr Steve Hambleton, called this situation “really disturbing”. The vaccination rate dropped both in low and high socio–demographic areas. (*Xinhua*, 11 Apr 2013)

• The HPV vaccination of young girls is showing successes in Australia. A study that compared rates of HPV–related diseases in the three years before the vaccine program and in the four years afterward in 86 000 persons showed a 93% decrease in genital warts in young women, although only 85% were vaccinated. This suggests that the herd immunity is protecting both young men and, in turn, unvaccinated women. (*New York Times*, 19 Apr 2013)

• Mr Bill Gates urged Prime Minister Julia Gillard to spend more foreign aid money on tackling malaria and polio during his visit to Australia in May this year. Mr Gates, who resigned from running Microsoft in 2009 to work full–time on his philanthropic activities, has steadily remained a driving force behind the GAVI Foundation, which has pledged to immunize 150 million people by 2015 in one of the largest population health projects that have ever been undertaken. (*news.com.au*, 7 May 2013)

• The Australian Government will increase its official development assistance to a record US$ 5.7 billion in 2013/14, an increase of approximately US$ 500 million (or 9.6%) on 2012/13, bringing the aid budget to 0.37% of gross national income (GNI). This will be the highest aid budget in Australian history, at a time when aid among OECD countries has fallen by 4% in real terms. Australia’s aim is to continue to increase its donations to 0.5% of GNI, deferring this target by one year – to 2017/18. (*Australian Minister for Foreign Affairs*, 13 May 2013)

## CHINA

• Within the first week into his tenure as China’s new president, Mr Xi Jinping already stressed his country’s strong and continuously growing ties to the African continent and the connected future of the world’s two developing economies. In recent years, China has played a major role in development of parts of sub–Saharan Africa and its economic importance in African region is steadily growing. (*Christian Science Monitor*, 19 Mar 2013)

• This year, 32 years since China’s National Population and Family Planning Commission (NPFPC) was established to oversee the world’s largest, longest–standing population control programme – the “one child policy” – the country’s leaders announced plans to dissolve it into the Ministry of Health. The National People’s Congress approved the proposal in March this year, and the commission ceased to exist, leaving the future of the country’s fertility policy in doubt. The “one–child policy” limited most Chinese families to a single child, and those in rural areas or those qualifying for other exceptions, to two, preventing more than 400 million births according to China’s national statistics. Financial Times reported that in the past 40 years Chinese doctors have performed more than 300 million abortions and about 200 million sterilisations. Many observers forecast a change in China’s fertility policy this year following the country’s once–in–a–decade leadership transition. For years, economists and demographers have argued that by constraining the size of the next generation, the country’s fertility policy has contributed to a grave demographic imbalance that could emerge in the coming decades, as the country’s elderly outstrip its labour force. (*The Lancet*, 23 Mar 2013)

• China’s “black clinics” are flourishing in big cities, as government debates health reform. Usually arranged as one–room shacks with a single light bulb on side streets, they provide the sole source of medical care for a growing population of migrant workers who are not recognized as city residents and do not qualify for cheaper healthcare at government hospitals. For many millions of migrant workers, whose hometowns are too far away to provide medical subsidizes, an unregulated world of back ally “black clinics” is the only option available. As the China’s economic growth depends on those workers, the two–tier nature of China’s overburdened health care system is beginning to appear inadequate to address this problem. A reform of the contentious “hukou” system of household registration may be required, although the system has been a cornerstone of government policy for decades. Dating back to 1958, the “hukou” system has split China’s 1.3 billion people along urban–rural lines. This prevented many of the about 800 million Chinese, registered as rural residents, from settling in cities and taking advantage of urban welfare and services. China’s new government is considering a change in this divisive system. (*Reuters*, 27 Mar 2013)

• The Health Effects Institute in Boston reported that more than 1 million people are dying prematurely every year from air pollution in China and presented their findings in Beijing. On many days in many cities, the air in China is thick with smog, with people commonly walking the streets wearing masks. It is now estimated that in China, approximately 1.2 million people die prematurely from exposure to outdoor air pollution, with about two–thirds of all the deaths now occurring in Asia. Air pollution has become the fourth leading cause of death in China, mainly affecting frail populations, such as people with asthma and the very young children who live in highly polluted areas. (*NPR*, 02 Apr 2013)

• The new H7N9 strain of the flu virus, first discovered in humans in March, has now killed at least 24 people. Cases of the virus were confirmed in more than 100 people and have spread to several new provinces in recent days, including Fujian and Hunan. In April, a man in Taiwan became the first case of the flu outside mainland China, having caught the flu while travelling in China. The World Health Organization has called the virus “one of the most lethal” and said that it was more easily transmitted than an earlier strain that has killed hundreds since 2003. Chinese scientists have confirmed that the bird flu strain has been transmitted to humans from chickens, but officials say there is no evidence yet of human–to–human transmission. (*Reuters*, 30 Apr 2013)

## EUROPE

• The debt crisis of the Euro zone continues, while austerity policies maintain the recession, according to an annual UN report. A press release, that announced the “World Economic Situation and Prospects 2013” report, produced by the UN Department of Economic and Social Development (DESA), the UN Conference for Trade and Development (UNCTAD) and UN regional commissions, highlighted that at least five economies are in recession, with very poor prospects for the Europe’s mid–term economic future. (*UN News*, 17 Jan 2013)

• Approximately 800 children in Europe have developed narcolepsy, a chronic incurable sleep disorder, after immunization with the H1N1 swine flu vaccine Pandemrix, manufactured by GlaxoSmithKline. Independent peer–reviewed studies in Scandinavia, Ireland, France and more recently in the UK demonstrate spikes in narcolepsy cases. The UK study published in the *BMJ* suggested a risk narcolepsy following Pandemrix vaccination of around one in every 55 000 doses. However, the lead author, Professor Liz Miller said: “Long term follow up of people exposed to Pandemrix is needed before we can fully establish the extent of the association.” (*Reuters*, 22 Jan 2013)

• At the World Economic Forum in Davos it has been announced that Germany agreed to provide 1 billion Euros to the Global Fund to Fight AIDS, Tuberculosis and Malaria, according to Germany’s Minister for economic cooperation and development, Mr Dirk Niebel. (*CNN*, 24 Jan 2013)

• Health officials in the UK said that a measles epidemic is “spreading at an alarming rate across areas of Wales”. The warning came as latest figures showed the number of cases in the Swansea area to more than double in less than a month, to 432 cases in total, of which 51 have been hospitalised. They added that, if the numbers of parents bringing their children for MMR jabs does not dramatically increase, measles will continue to spread, predicting that the outbreak could result in about 1000 cases. (*BBC*, 26 Mar 2013)

• French Health Ministry said that France has identified its first case of a new strain of coronavirus, emerging from the Middle East, in a person recently returned from a stay in the United Arab Emirates. The virus came to scientists’ attention in September 2012. Since then, there have been at least 30 laboratory–confirmed infections with the virus globally, including 18 deaths, according to the World Health Organization. (*Reuters*, 8 May 2013)

## INDIA

• Recent advances in Chinese vaccine manufacturing are beginning to threaten India’s supremacy in this market. Currently, India produces 40–70% of the World Health Organization’s demand for DPT and BCG vaccines and 90% of measles vaccines, exporting them to 150 countries. Mr PV Appaji, Director General of the Pharmaceuticals Export Promotion Council of India stated that the government should come forward to help Indian vaccine makers with capacity building by offering funds at affordable interest rates, adequate power supply and infrastructure. He added that new export markets for Indian pharmaceutical industry were being identified, including Latin America and Africa, and that Indian pharmaceutical export target of US$ 25 billion by 2014 had now been revised to 2015 due to weak international economic situation. (*Times of India*, 20 Dec 2012)

• In January 2013, the Indian government launched an innovative direct cash transfer program that deposits government pension and scholarship payments directly into recipients’ bank accounts. Despite being currently operative in only 20 districts, the program is seen as a leap forward for India’s antipoverty agenda, as the current practices often inadvertently result in feeding corrupt intermediaries instead of intended recipients. However, critics assert that the program is simply a political ploy to buy the votes of the poor, and that in a country where millions do not even have access to banks, direct money transfers are only a minor aspect of a much greater effort needed to permanently lift people out of poverty. This includes provision of better schools, hospitals and shops. (*New York Times*, 05 Jan 2013)

• There are Indians who welcome the end of British aid for India, announced for 2015, on grounds of national pride. However, Amanda Glassman, director of global health policy at the US–based Centre for Global Development, acknowledged that British aid for India was small in absolute terms, but said it could still make a big difference, especially in India’s poorest states where only 44% of children under five are fully vaccinated. (*The Guardian*, 18 Feb 2013)

• India’s Supreme Court rejected Novartis AG’s attempt to patent a new version of a cancer drug in a landmark decision. Novartis argued it needed a patent to protect its investment in *Glivec *(imatinib), used in treating chronic myeloid leukaemia and other cancers. However, campaigners argued that the company was trying to use loopholes to make more money out of a drug whose patent had expired. This decision sets a precedent that will prevent international pharmaceutical companies from obtaining fresh patents on updated drugs which makes them unaffordable for most of India’s 1.2 billion population, where 40% earn less than US$ 1.25 a day. Healthcare activists claim that this will ensure that poor patients around the world would continue to get access to cheap generic versions of the lifesaving medication. *Glivec* costs about US$ 2600 a month, whilst the generic version in India costs US$ 175 a month. The generic version produced in India makes it accessible to poor not just in India, but also across the world. (*The Guardian*, 1 Apr 2013)

• Indian Department of Biotechnology plans to build on the success of the public–private partnership that led to the development of the first indigenous rotavirus vaccine to combat childhood diarrhoea. The Department announced its ambition to shift its target towards studying vaccines for dengue, tuberculosis and malaria, with a particular focus on affordability. The Department will readily co–operate with the pharmaceutical industry in the development of future vaccines. (*The Hindu*, 16 May 2013)

## THE AMERICAS

• The international response to the earthquake in Haiti was overwhelming: more than US$ 9 billion was raised from public and private donors. Official bilateral and multilateral donors pledged as much as US$ 13 billion and almost 50% of these pledges have been disbursed, according to UN sources, while private donations added further US$ 3 billion. However, three years after the tragedy it is not really clear how the money was spent or whether the desired outcomes were achieved. (*The Guardian*, 14 Jan 2013)

• Leaving the post of the US secretary of state, Ms Hillary Rodham Clinton will be remembered for not concentrating on long–term intractable conflicts, but rather focusing on more feasible international development goals: saving children from dying of preventable diseases, connecting poor farmers to markets as part of a new food security program, and improving the prospects for women who lack of education and resources. During her tenure, she became an icon of global development. (*Devex*, 31 Jan 2013)

• GlaxoSmithKline Plc (GSK) and the Texas A&M University System won approval from the US Health and Human Services Department for a US$ 91 million flu vaccine plant. The facility would be able to produce treatments in response to pandemics or biological attacks. It should be located in the College Station, Texas, northwest of Houston, creating jobs for up to 300 people directly, but up to 7000 further indirect and related jobs. (*Bloomberg*, 26 Mar 2013)

• The joint study between United Nations Children’s Fund (UNICEF) and the Mexican Government reported that more than 20 million children and adolescents in Mexico are estimated to live in poverty, with five million in extreme poverty. The UNICEF Representative in Mexico, Ms Isabel Crowley, said that although the economy has grown well in Mexico, this does not always mean that the poor are better off, because human development indexes in parts of Mexico are close to those of some of the world’s least developed countries. Moreover, children are overrepresented among the poor. In 2010, 46% of all Mexico’s residents lived in poverty, but in children the poverty rate rises to 54%. The study also reported that nearly 14% of Mexican pre–school children are stunted, ie, slowed in their development, which is often a result of malnutrition. The rate is higher in rural areas, reaching nearly 33% among indigenous children. (*UN News*, 3 Apr 2013)

• The Information Technology and Innovation Foundation (ITIF), a US–based think tank, recommended that the Congress should suspend trade benefits for India. This comes as a result of growing unease in the US over Indian policies that block American exports, and their exploitation of costly US research to develop new medicines and other forms of valuable intellectual property. The US House of Representatives’ Ways and Means Committee recently held a hearing on this topic, where a larger number of complaints were made about India’s industrial policies. ITIF listed actions and policies taken by the Indian government that were “stripping” foreign biopharmaceutical companies of valuable patent protections. (*Reuters*, 14 May 2013)

**Contributors:**

Rachel Banfield, Frances Barclay, Vijna Hiteshna Boodhoo, Katherine Booth, Rachel Burge, Michael Charteris, Alexander Fullbrook, Jenny Hall, Ewan D. Kennedy, Nethmee S. Mallawaarachchi, Diana Rudan, Victoria Stanford, Goran Zangana

